# Cochlear Implantation After Temporal Bone Fracture: A Systematic Review of Preoperative Predictors and Timing

**DOI:** 10.3390/brainsci16020227

**Published:** 2026-02-14

**Authors:** Elias Antoniades, George Psillas, Parmenion P. Tsitsopoulos, John Magras, Petros D. Karkos

**Affiliations:** 1 Second Department of Neurosurgery, Aristotle University School of Medicine, 54636 Thessaloniki, Greece; 2 First Academic ENT Department, Aristotle University of Thessaloniki, AHEPA Hospital 1, Stilponos Kyriakidi St., 54636 Thessaloniki, Greece; psill@otenet.gr (G.P.); pkarkos@aol.com (P.D.K.)

**Keywords:** cochlear implantation, temporal bone fracture, post-traumatic deafness, preoperative assessment

## Abstract

**Highlights:**

**What are the main findings?**

- Cochlear implantation is a viable treatment option after temporal bone fractures when the cochlear nerve is anatomically intact.
- Preoperative radiological and electrophysiological assessments are crucial for candidates’ detection and surgical strategy.
- Most included studies exhibit meaningful speech benefit.

**What are the implications of the main findings?**

- Timely implantation limits post-traumatic cochlear ossification and may result in improved outcomes.
- Trauma-related sequels are not so frequent and do not preclude successful placement and outcomes.
- Cochlear implantation, based on these limited observational data, is preferred over Auditory Brainstem Implantation.

**Abstract:**

**Background/Objectives**: Cochlear implants (CIs) constitute a viable method for auditory rehabilitation in patients with profound sensorineural hearing loss after temporal bone fractures (TBFs). These patients comprise a challenging population due to the anatomical deformity and neural injury. **Methods**: By performing this systematic review, we attempted to evaluate the viability of CIs in the context of TBF. The literature search, across Pubmed/MEDLINE, Scopus and Google Scholar, was performed under the PRISMA guidelines. The selected time period was from December 1995 to September 2025. The final analysis included 11 manuscripts. The majority of the studies were retrospective case series with a moderate risk of bias. **Results**: The primary outcome was postoperative auditory function, evaluated with speech perception tasks and aided sound-field pure-tone audiometry. The secondary outcomes were the report of radiological and electrophysiologic prognosticators of implants’ viability, timing of surgery, procedural feasibility and complications. Across the studies, CIs conferred meaningful auditory benefit when the cochlear nerve was intact. High-Resolution Computed Tomography (CT) was utilized for TBF classification and cochlear patency, whereas Magnetic Resonance Imaging (MRI) and a promontory test were crucial for the assessment of neural integrity. Prompt placement, optimally within 12 months after trauma, was related to improved outcomes by limiting cochlear fibrosis and ossification. Despite patients’ impedance fluctuation, restricted speech perception in noise and frequent abnormal facial nerve excitation, the overall audiologic and speech discrimination results are comparable to non-trauma recipients. **Conclusions**: A CI appears to be the choice of treatment over auditory brainstem implants, as long as the cochlear nerve remains intact. Rapid implantation in well-selected patients coupled with ordinal mapping and follow-up can restore dysfunctional hearing and improve patients’ quality of life.

## 1. Introduction

Temporal bone fractures (TBFs) constitute a subset of craniofacial trauma and account for approximately 18–40% of skull base fractures. [1]. TBFs propagate along predictable anatomical pathways. The majority do not violate the otic capsule and traverse the mastoid process or external auditory meatus. Otic capsule-violating fractures extend to the cochlea, vestibule or their junction, a scenario which strongly relates to sensorineural hearing loss [1]. Auditory augmentation involves many procedural techniques when classical air conduction hearing aids are not beneficial. Bone-anchored hearing aids (BAHAs) are applicable in conductive and mixed auditory impairment. Their advantageous effects in sound perception, speech discernment, and quality of life are documented [2]. Patients, though, with progressive sensorineural hearing loss may ultimately require transition to cochlear implantation. This fact stresses their limited role when cochlear or neural function is compromised [2].

Transcutaneous active bone-conduction systems exhibit benefits in pure-tone audiometry (PTA) and speech discernment, with lower percentages of system malfunction [3]. Their prerequisite is, though, a minimum of cochlear function. Therefore, they do not provide auditory rehabilitation in patients with profound hearing loss or deafness [3].

On the contrary, cochlear implants (CIs) excite the cochlear nerve and contribute to acoustic rehabilitation, particularly in cases of bilateral severe sensorineural hearing loss (SNHL) [4]. Their potential disadvantages are the reliance on the grade of neural integrity and cochlear ossification. They are more effective in the field of social interaction and speech intelligibility compared with alternative bone conduction devices [4]. Despite the utilization of CIs for severe-to-profound hearing loss, their contribution to TBFs is still poorly defined. Post-traumatic cochlear deformation, insidious ossification and cochlear nerve impairment render patients’ selection and timing of surgery complicated. We conducted this study to synthesize the existing data in a systematic way, focusing on the preoperative parameters, timing considerations and audiological outcomes.

## 2. Materials and Methods

This systematic review was conducted and reported according to the Preferred Reporting Items for Systematic Reviews and Meta-Analyses (PRISMA) guidelines [5]. The completed PRISMA 2020 checklist is included as Supplementary Material S1. The review protocol was not registered. The MeSH words that were used were (Hearing Loss) AND ((Head Trauma) OR (traumatic brain injury)) AND (Hearing Aids). We intentionally selected broader MeSH terms as a means to optimize the sensitivity of the literature search. Both “temporal bone fractures” and “cochlear implants” terms are subsumed under more extended categories, such as head trauma or traumatic brain injury and hearing aids, respectively. Consequently, relevant studies were likely to be omitted. Fracture-related sequels and cochlear implants insertion effects were applied as inclusion criteria across the screening of titles, abstracts and manuscripts. Complete electronic search procedures for each database are reported in Supplementary Materials S2. The final search was performed on 30 September 2025.

We excluded studies that did not refer to human subjects or post-traumatic hearing loss. In addition to this, we did not include studies that did not involve cochlear implantation or those that lacked detailed postoperative data. Finally, cadaveric or single case reports and non-English publications were precluded, as well.

**Figure 1:**
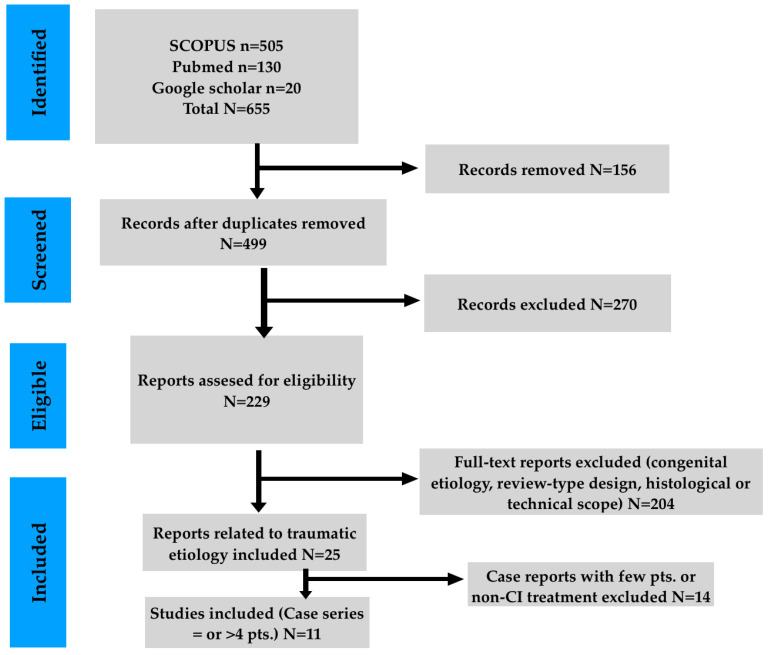
PRISMA flowchart.

We included studies from December 1995 until September 2025. The literature search was conducted in PubMed/Medline, Scopus and Google Scholar. Two reviewers independently scanned the titles and summaries for relevance to the insertion of CIs in human subjects. Because Google Scholar does not provide full Boolean reproducibility, its use may bear selection bias. In our attempt to address this issue, we screened the first 200 results, based on their relevance, and verified all eligible studies against Pubmed and Scopus. The initial identification conferred 655 records. After extracting duplicates, 499 sole manuscripts were detected and evaluated for eligibility. Records that were not related to human individuals, were anatomical studies on cadavers or were irrelevant to CI insertion were removed. Thereafter, 229 full-text reports remained for eligibility appraisal. Following PRISMA 2020 guidance, the argumentation for studies’ removal was predefined and systematically applied. Of these, 204 reports were excluded due to congenital etiology, review-type design, or histological/technical focus. Because multiple exclusion criteria usually applied to the same report, numerical itemization was not feasible. The remaining 25 reports underwent further assessment, and 14 were excluded because they did not involve cochlear implantation or included fewer than four patients. The latter was the attempt to maintain methodological strictness. Finally, studies without multimodal audiologic and speech discrimination protocols were excluded, as well. Disparity in records’ inclusion was amended via consensus. Hence, we concluded with 11 clinical series. The PRISMA 2020 flow diagram is presented in Figure 1. The studies finally included in the analysis were observational case series involving a limited number of participants. The texts of these records were assessed, focusing on the CI procedure in the context of TBF or post-traumatic auditory impairment. Parameters that were evaluated were the epidemiological data of the patients, TBF type, timing of surgery, intraoperative findings, audiological and speech perception outcomes, and complications, as well.

Quantitative synthesis was not feasible due to the significant heterogeneity in audiological outcome tools and patients’ observation duration. Different speech perception tests are not comparable, and reporting formats are inconsistent. Hence, calculation of pooled effects estimates or heterogeneity metrics like I^2^ was methodologically precluded. For this reason, our synthesis remained narrative.

Screening through the included reports on CIs in cases of TBF revealed the re-establishment of significant hearing performance when the eighth cranial nerve is intact. Computed tomography (CT) was used in all studies for TBF diagnosis and classification. Magnetic Resonance Imaging (MRI) was used to detect the integrity of the scala tympani. Promontory stimulation was utilized to confirm the functional performance of the cochlear nerve. Free-field audiometry reveals the lowest intensity threshold that the patient perceives or the speech perception at a given intensity. When a CI processor is used for this test, the test is mentioned as aided pure-tone audiometry. Closed-set tests (wherein patients choose the correct answer) estimate basic speech intelligibility. On the contrary, open-set tests (wherein patients repeat words or sentences that they just heard) evaluate real speech perception.

- Outcome measures

The primary outcome of the systematic synthesis was postoperative audiological performance after CI placement in the context of TBF. The evaluation took place with speech-perception (open- and closed-set tasks) and aided sound-field pure-tone audiometry. Secondary outcomes were assessed by electrophysiologic correlates of cochlear nerve viability, the procedure’s timing, surgical feasibility, postoperative sequels and mapping consistency.

- Risk-of-bias evaluation

All included manuscripts were categorized as retrospective observational studies, which de facto respond to level IV evidence. The Joanna Briggs Institute (JBI) Case Series Checklist was used to measure the risk of bias of these 11 manuscripts [6] [Table 1]. Small case series, with fewer than four patients, were regarded as analytically equivalent to case reports of significantly poor evidence and liable to selection bias. This tactic conforms to the JBI methodological process, which presupposes the inclusion of multiple individuals for clinical patterns detection and anecdotal bias reduction. Consequently, the ≥4-patient threshold was adopted within the included evidence base [6]. Some included reports were comparative analyses, but these were descriptive and exploratory in nature. Furthermore, they did not involve predefined control groups, and their aim was not to estimate the causal intervention impact. For all these reasons, risk-of-bias tools targeting non-randomized comparative intervention studies were methodologically not applicable.

**Figure 2:**
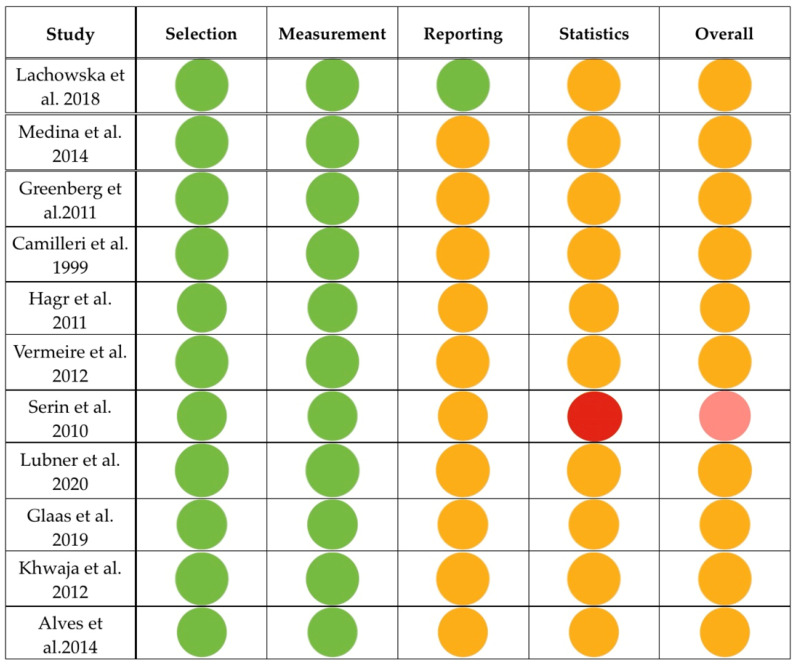
Traffic-light summary of overall risk of bias across included studies. Risk of bias was evaluated using the Joanna Briggs Institute Case Series Checklist and grouped into 4 domains (selection, measurement, reporting and statistical bias). Green light: low risk, yellow light: moderate/unclear risk, and red light: high risk, Pink light: moderate to high risk [7,8,9,10,11,12,13,14,15,16,17].

Owing to the descriptive character of the manuscripts, no study met the criteria for low risk of bias. Most of the manuscripts revealed a moderate risk, which was expected in observation studies of infrequent trauma events. In addition, the manuscripts were retrospective studies, which carry intrinsically sampling bias. Consecutive inclusion (Q4) was not documented in any of the manuscripts and, consequently, was qualified as negative. Classic statistical tasks could not be applied in the population of any study due to the small sample sizes, and, therefore, the quality of statistical analyses (Q9) was classified as negative, as well. Only one manuscript by Serin et al. manifested a moderate–high risk owing to the preoperative selection of patients (the ones that had intact 8th nerve) and the small number of participants who were not contrasted with a control group [13]. The rest of the articles carried a moderate risk. Furthermore, none of the studies had a standardized protocol of patients’ inclusion anwd follow-up control. Despite these limitations, diagnostic approaches and outcome evaluations were ratified and suitably utilized. We have added a simplified traffic-light summary to provide a rapid visual assessment of the overall risk-of-bias patterns (Figure 2).

## 3. Results

The eleven included manuscripts in the final synthesis manifested significant heterogeneity regarding the studies’ design, patients’ selection, type of TBF, timing of surgery and audiologic follow-up. Several common parameters could be, though, revealed, which allowed a ratified comparison among the manuscripts. This was based on the indications and work-up of surgery, the temporal interval between the trauma and the operation and the duration of the postoperative audiological observation, as well (Table 2).

- Frequent complications of cochlear implants (Table 3)

The major complications across the studies were the abnormal facial nerve stimulation, the ossification of the cochlea and the partial placement of the electrode. Six studies had the manifestation of facial nerve excitation. Among these, only Khwaja et al. referred to “otic capsule involvement” [16]. The remaining studies by Camilleri et al. [10], Serin et al. [13], Greenberg et al. [9], Lubner et al. [14] and Glaas et al. [15] used equivalent terms, such as “transverse fractures” and “crossing of vestibule or basal turn or promontory”. Glaas and his colleagues presented six patients with CIs who exhibited constant audiological improvement (50% at 6 months and 67% at 12 months). One out of six patients had facial nerve aberrant stimulation, implying that this sequel does not preclude the overall rehabilitation of the cochlear nerve [15].

Cochlear ossification was reported in seven studies, as well. Greenberg et al. estimated the rate of labyrinthitis ossificans at 17.6% in their group, which was detected with HRCT [9]. Medina et al. reported three patients in their group with cochlear obliteration (approximately 20%). Considering that the rate was significant, they recommended prompt control after patients’ stabilization [8]. Serin et al. [13], Lubner et al. [14] and even Camilleri et al. [10] since 1999 supported the preoperative assessment of ossification with both CT and MRI, which constitutes a significant obstacle for complete electrode placement. Khwaja et al. indicated the basal turn as the starting point of the phenomenon [16]. Finally, Alves et al. were more descriptive regarding the length and the grade of ossification, which indeed initiates from the basal turn [17]. Hagr et al. stressed the joint use of HRCT and MRI preoperatively, and, therefore, they were able to avoid this situation in their group. They stressed that HRCT may misdiagnose luminal obstruction, which is usually noticed intraoperatively [11]. None of the studies, though, used combined numerical data as contraindications for CIs or focused on surgical nuances as candidacy criteria.

From the studies included, Serin et al. [13], Camilleri et al. [10] and Khwaja et al. [16] reported the surgical impact of ossification, which was partial insertion. Together with them, Lubner et al. [14] stated that in four out of 24 (17%) CI recipients, the insertion was partial. CT imaging in all series was performed in the context of the preoperative workup, thus, late from the traumatic event, and when cochlear deformity was established.

- Audiological and speech perception outcome (Table 3)

The pure tone audiometry observational protocol was mentioned elaborately in three publications. Lachowska et al. used PTA after the device’s insertion at 0.5, 1, 2, and 4 kHz. The authors reported a constant audiological improvement until the last follow-up at the first year. They did not report, though, dB values numerically, but rather graphically [7]. Alves et al. presented their audiological results with details. These involved mean thresholds of approximately 33.3 dB at 500 Hz, 33.3 dB at 1000 Hz, and 35.8 dB at 4000 Hz in the patients with TBF. These were similar to the non-trauma recipients [17]. Finally, Vermeire et al. referred to PTA results counting approximately 28 dB HL at 0.5–4 kHz, which indicates that tonal access in post-traumatic ears can reach levels typical of successful adult cochlear implantation [12].

The remaining studies focused mostly on speech perception tasks. The first reported group by Camilleri et al. [10] achieved sufficient open-set speech perception, despite the adverse effects of FN stimulation. The results were moderate in fluctuating sound frequencies and noisy environments [10].

Serin et al. documented successful open-set three-syllable word apprehension of >90% and telephone utility in the first year in most of their group. Only in one patient with progressive cochlear ossification did perception not exceed 20% [13]. Similar findings were recorded by Lubner et al., who evidenced poorer word perception under ossification in noise. This difference had no statistical significance, though [14]. In the greatest trauma group, Khwaja and his colleagues reported moderate outcomes. Their results were heterogeneous, and the test they used did not target the rapidly alternating high frequencies (the Bamford–Kowal–Bench and City University of New York tests) [16]. Even in these tests, their patients were far from excellent performers. The authors revealed, though, that non-TBF trauma patients may also suffer profound SNHL and can benefit partially from CIs [16].

The study by Vermeire et al. demonstrated great variability in the outcomes. Speech-in-noise results were rather good only for one patient, but the rest of the outcomes were either unmeasurable or extremely low. Interestingly, in all patients, the promontory test was normal, which could not constitute a predictor for the outcome. Another paradox was the dissociation between audiological performance and quality-of-life outcome [12]. In these patients, self-perceived handicap referred to emotional burden, compensating tactics (such as lip reading or contextual understanding) and anticipations. Consequently, very good responders reported profound handicap, while partial responders reported moderate to slight satisfaction. Another important aspect is the fact that the post-implantation period is actually a state of latent hearing loss with impaired perception in a noisy environment [12].

Overall, numerical success rates could not be reliably extracted because outcome measures, definitions of success, and follow-up durations varied significantly across studies. Therefore, outcomes were presented descriptively [Table 3].

## 4. Discussion

In this study, we focused on the long-term viability, outcomes and complications of CIs in TBF patients. Throughout the included manuscripts, CIs conferred noticeable acoustic rehabilitation in selected patients, especially when the anatomical and functional integrity of the cochlear nerve was preserved. The adverse effects in trauma cases revealed a similar incidence compared with non-trauma cases [6].

Post-traumatic SNHL is characterized primarily by impaired speech discrimination and secondarily by elevated PTA thresholds [18]. It is attributed to both cochlear nerve injury and cochlear deformation, as a result of fibrosis, ossification and discontinuation of fluid compartments [6]. Consequently, both temporal and spectral sound processing are significantly affected [19].

The integrity of the cochlear nerve confirmed by MRI T2 sequences, positive promontory testing and prompt insertion, preferably within the first year, are considered major positive prognosticators [20]. Electronystagmography was integrated into the preoperative setting in many studies. Although it does not evaluate the function of the cochlear nerve, it provides adjunctive information on the vestibular nerve and the labyrinth’s integrity. Cognitive deficits after head injury affect auditory rehabilitation and necessitate prolonged mapping periods [21]. These handicaps of post-traumatic SNHL can be ameliorated by CIs [22]. The generalizability of CI outcomes in these patients should be interpreted cautiously, as periprocedural and post-traumatic complications may have a negative effect on patients’ outcomes and the device’s performance.

Abnormal facial nerve (FN) stimulation is mainly attributed to the aberrant electrical current spread through the low-impedance corridors of the fractured bone [6,17]. Across all included studies, FN stimulation did occur more frequently in post-traumatic recipients, which was amenable, though, via adjustment of the stimulation’s levels and pulse width or selective electrode deactivation [23,24]. Elevated electrode impedance, which is marked during the healing process (after the 6th week), may further contribute to abnormal FN stimulation. Fortunately, the system’s explantation is rarely necessary, and auditory rehabilitation is not hindered [6].

Cochlear fibrosis and ossification constitute major limiting factors, especially when a fracture’s line transects the cochlea. They are more prominent at the basal curve and round window and extend to the scala tympani [16,25,26]. Across the included studies, advanced post-traumatic ossification was related to complicated implantation, incomplete electrode insertion, and suboptimal speech perception outcomes. Many authors advocate for prompt insertion before osteogenesis [7,13]. In these scenarios, an alternative cochleostomy at the scala vestibuli or second entry point rostral to the oval window may be opted for. The results are not so fruitful, though [19]. When the cochlea is totally occluded or the cochlear nerve is proven non-functional, auditory brainstem implants (ABIs) are a viable alternative. The rationale is that an ABI circumvents both the cochlear nerve’s emerging point and trunk [27]. Finally, the device’s failure may appear late and respond to electrode transposition in the context of labyrinthine deformity. Cochlear lateral wall disruption, violation of the scala tympani and waning of the spiral canal/modiolus promote the deficiency of the basilar membrane and increase the risk of electrode translocation [28,29,30].

Despite satisfactory aided pure-tone thresholds, reduced speech perception in noise remains in CI recipients after TBF [31]. The major problem is the spectral frequency overlapping of consonants and misuse of intonation signals [32]. Patients make an exaggerated effort in a multi-talker ambience, even when the device status appears acceptable [33]. Suboptimal outcomes are detected by open-set speech perception. This phenomenon is acknowledged as hidden hearing loss (HHL) [33,34]. The latter refers to the auditory dysfunction, which relates to normal PTA thresholds with significant low-spontaneous-rate cochlear nerve fibers depletion, which is actually the hallmark of synaptopathy [35]. Therefore, sound is detected, but higher-order speech discrimination remains restricted [36].

Hagr and his colleagues characteristically reported an overall good outcome in closed-set tasks, but not a similar one in open-set tasks [11]. This characteristic dissociation delineates the primary therapeutic aim of CI placement after TBF, which is the preservation of sound discrimination, rather than its detection alone. Greenberg and his colleagues revealed problematic speech discrimination, especially in monosyllabic word recognition, which is attributed to the frequencies overlapping [9].

Glaas et al. [15] employed a robust preoperative work-up, which involved transmission-evoked otoacoustic emissions (TEOAEs), brainstem-evoked response audiometry (BERA) and the promontory test. In this way, they confirmed if the lesion was indeed cochlear (absent TEOAEs) and documented the nerve’s functional intactness (a positive promontory test and absent BERA). They achieved this by recording both the efficiency of outer hair cells and the conductivity of the 8th nerve. In this way, they clearly presented OAE-ABR dissociation as a qualifying and novel approach for detecting neural dysfunction consistent with what is now recognized as synaptopathy.

In any case, CI follow-up should be held periodically within the first twelve months, including frequent mapping [34]. Electrically evoked responses reveal the frequency shifting. Fortunately, the removal of the device is necessary in rare cases. The risk is greater when the basal turn array is dysfunctional. Therefore, in incomplete electrode placement, less aggressive stimulation at lower frequencies is a rational tactic that prevents overactivation of residual neurons [37].

The classic PTA, with headphones and bone oscillators, is designed to examine the normal auditory pathway and not the circumvention that is mediated by a CI. From the context of the manuscripts of Lachowska et al. [7], Vermeire et al. [12] and Alves et al. [17], we assume that these tests were actually aided sound-field pure-tone thresholds. The authors’ attempt was to obtain clear and objective values of acoustical minimal effective performance at speech-related frequencies (0.5–4 kHz) and to compare them with the preoperative ones. They also used these results to verify the device’s adequate stimulation and as a baseline for further mapping. This assessment tool, though, cannot independently evaluate the suprathreshold speech analysis.

As far as the surgical candidacy is concerned, the radiological preoperative work-up contributes significantly. The innate parameters of the cochlea are predictors of beneficial implantation. When the rate of cochlear duct narrowing in the first 45 degrees exceeds 24.5%, then the translocation risk is considered high [38]. The modiolar array tends to translocate more frequently compared with the lateral wall one (43% vs. 7%). The latter one, on the contrary, exhibits lower rates of tip fold-overs. Therefore, High-Resolution CT evaluation should also focus on modiolar base defects, which relate significantly to CSF gusher, as well. Apart from these, the route of the FN should also be estimated [39].

On MRI and especially T2 sequences, a normal acoustic nerve caliber counts almost 1.8 mm at the porus acousticus and 1.2 mm at the middle of the internal acoustic canal. Nerve areas of <0.7 mm^2^ in any slice on High-Resolution MRI relate to poor rehabilitation [40,41]. On the contrary, cochlear patency or modiolar integrity is described qualitatively [42]. Additionally, the deficient pneumatization of mastoids, the narrowness of the facial nerve recess (<3 mm) and the high-riding course or dehiscence of jugular bulbs render insertion laborious [43]. Finally, the angular course of the seventh nerve is also an independent criterion of successful placement [44]. As a whole, these parameters may be used as secondary considerations in the surgical planning of TBF cases, as well.

In cases of traumatic nerve compression and complete cochlear ossification, ABI may take place. According to Colletti et al., after ABI placement, speech recognition in both closed- and open-set sentences reaches 100%. Therefore, ABIs constitute a reasonable second-tier option [45]. They warrant, though, more delicate and long-term mapping. The lack of tonotopic organization of the cochlear nucleus, its proximity to other brainstem nuclei and patients’ satisfaction are crucial factors for the outcomes [33].

Medina and his colleagues provided robust data for the higher-ranking performance of CIs compared with ABIs in TBF patients. Patients exhibited better speech perception in both open- and closed-set tests. ABIs have an unstable course, which involves the risk of open circuits and non-auditory responses, which may warrant the shutdown of the electrodes. Therefore, based on this limited comparative observational evidence, CIs remain the treatment of choice in post-traumatic scenarios [8].

**Figure 3:**
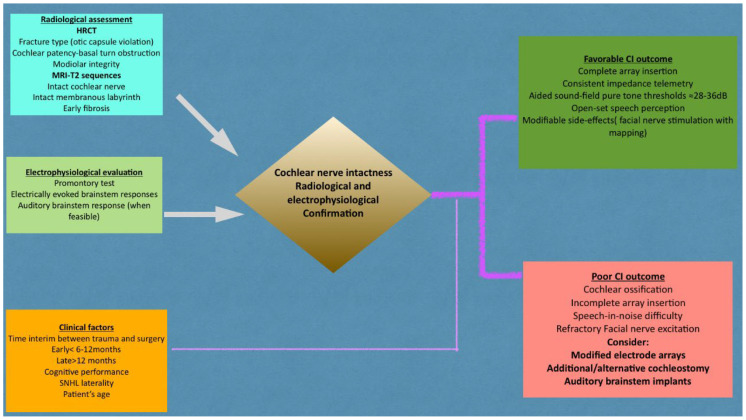
Integrated decision-making pathway for cochlear implantation after temporal bone fractures. All imaging, electrophysiological and clinical evaluations converge on detection of cochlear nerve’s intact status. This constitutes, actually, the prerequisite for the procedure’s viability. Good outcomes relate to complete array insertion, non-fluctuating impedance telemetry and significant speech perception. On the contrary, late placement, cochlear ossification and neural injury may not confer favorable outcomes and warrant alternative surgical methods, including modified electrode arrays or auditory brainstem implants. Converging arrows represent the integration of multimodal assessments. The thicker pathway denotes the principal decision-making trajectory, while thinner arrows indicate contributory evaluation steps.

The integrated clinical, imaging, and electrophysiological steps conferred by the current analysis are summarized in Figure 3.

Our study, hence, constitutes a systematic synthesis of an overall rare clinical scenario, combining radiological, electrophysiological, surgical and audiological data. The aim was the clarification of CI candidacy after TBF. Eastwood et al. [6] published a foundational, outcome-focused review of CIs following TBF. Building on this work, the current review extends the literature by highlighting the timing of surgery. It also incorporates preoperative imaging and electrophysiologic parameters, which verify cochlear nerve viability. Additionally, the interpretation of outcomes takes place within a synaptopathy-based framework.

The explicit comparison with alternative rehabilitation treatments and the highlighting of trauma-related sequels provide applicable guidance for evidence-based decisions in post-traumatic SNHL.

## 5. Limitations

This review has its limitations. Despite its systematic character, pertinent manuscripts located in other databases may have been missing. In addition to this, extracting results from Google Scholar is not a completely reproducible process. Furthermore, only a search in the English language was performed, and that may bear bias. Importantly, the manuscripts responded to small observational and retrospective studies. This means that no included report met the criteria for low risk of bias, and causal inferences cannot be drawn with certainty. Consequently, the findings should be regarded as hypothesis-generating and not definitive.

Furthermore, all included studies had significant methodological discrepancies both in the timing of placement and in the presentation of outcomes. Therefore, the robustness of the conclusions is restricted and makes meta-analysis extremely difficult.

## 6. Conclusions

Cochlear implantation is an applicable and efficacious procedure for post-traumatic profound hearing loss or deafness, as long as the cochlear nerve is intact. Radiologic work-up with High-Resolution CT and T2-Weighted MRI, combined with electrophysiologic trials, such as promontory test and electrical auditory brainstem responses, constitutes the gold standard for patient selection. Prompt placement (<12 months) is associated with improved outcomes by avoiding continuous fibrosis and ossification, which may hinder electrode insertion and reduce functional outcomes. Even though patients with TBF may manifest frequent impedance fluctuations, occasional facial nerve stimulation, and decreased speech discrimination at high frequencies, postoperative results bear similarities to those of non-traumatic cochlear implant recipients. These observations render cochlear implants a viable rehabilitation tool in selected TBF patients, stressing the significance of rapid implantation and tailored mapping for each patient.

## Figures and Tables

**Table 1:** Risk-of-bias evaluation for the included manuscripts. Q1: clear inclusion criteria, Q2: condition measured in a standard, reliable way, Q3: valid identification methods, Q4: consecutive inclusion, Q5: complete inclusion of participants, Q6: demographics stated clearly, Q7: clinical information presented clearly, Q8: clearly reported outcomes, Q9: appropriate statistics, and Q10: follow-up complete/clearly reported. Green light: low risk; yellow light: moderate/unclear risk; red light: high risk; Pink light: moderate to high risk.

Study	Q1	Q2	Q3	Q4	Q5	Q6	Q7	Q8	Q9	Q10	Overall	Risk
Lachowska et al. [7]	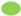	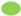	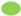	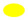	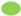	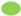	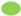	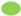	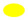	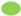	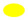	Moderate
Medina et al. [8]	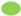	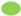	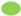	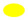	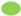	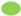	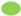	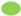	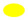	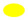	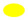	Moderate
Greenberg et al. [9]	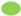	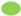	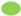	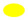	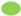	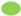	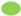	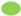	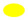	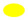	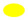	Moderate
Camilleri et al. [10]	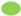	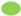	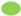	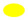	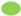	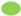	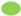	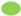	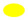	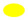	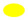	Moderate
Hagr et al. [11]	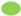	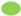	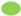	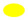	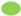	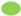	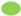	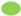	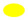	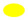	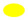	Moderate
Vermeire et al. [12]	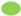	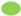	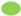	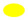	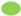	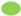	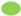	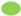	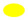	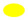	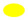	Moderate
Serin et al. [13]	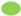	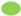	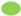	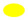	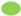	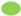	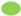	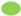	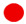	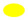	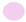	Moderate-high
Lubner et al. [14]	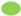	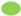	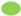	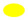	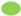	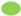	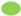	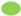	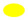	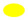	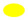	Moderate
Glaas et al. [15]	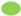	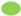	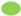	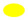	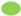	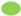	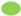	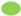	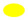	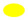	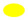	Moderate
Khwaja et al. [16]	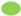	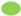	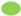	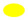	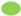	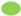	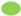	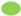	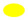	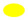	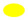	Moderate
Alves et al. [17]	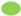	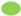	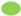	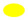	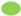	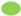	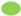	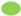	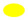	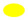	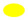	Moderate

**Table 2:** Major characteristics of the included studies concerning CIs for post-traumatic hearing loss in the context of temporal bone fractures (AB: Arthur Boothroyd, ABI: auditory brainstem implant, ABR: auditory brainstem response, BERA: brainstem-evoked response audiometry, BKB: Bamford–Kowal–Bench, CT: computed tomography, CUNY: City University of New York, MRI: Magnetic Resonance Imaging, EABR: electrically evoked auditory brainstem response, ENG: electronystagmography, HL: hearing loss, PTA: pure-tone audiometry, SNHL: sensorineural hearing loss, TBF: temporal bone fracture, and VEMP: vestibular-evoked myogenic potentials.

Name	Number of PTs and CIs	Major Indications	Interval Between Trauma and Implantation	Follow-Up Intervals	Preoperative Assessment
Lachowska et al., 2018 [7]	7 patients 7 CIs	Profound SNHL Intact cochlear nerve and patent cochlear duct	5.77 months [0.8–6.73 months]	Upon the 1st and 12th months; then, annually up to 16 years	PTA, speech perception, ABR, and caloric testing CT and MRI (not always)
Medina et al., 2014 [8]	11 patients 8 CIs 3 ABIs	Severe to profound SNL CI if cochlear nerve intact based on EABR ABI otherwise	Months to several years	From 1 to 10 years; initially at the 1st, 3rd, 6th and 12th months and then annually	PTA, speech perception, ABR and EABR Promontory test and ENG CT and MRI
Greenberg et al., 2011 [9]	25 patients 11 CIs (unilateral) (7 bilateral and 4 unilateral TBFs)	Severe or profound SNHL Intact cochlear nerve	1 month–9 years	Upon the 12th month; then, prolonged observation and, in some cases, up to 20 years	PTA, promontory test, and ENG CT
Camilleri et al., 1998 [10]	7 patients with bilateral TBFs 7 CIs	Profound SNHL > 90 dB Intact cochlea	Months to a few years	Upon the 1st month and the 9th month; overall observation period was 9 months	PTA, speech audiometry, promontory test ABR (some patients) CT and MRI (some patients)
Hagr et al., 2011 [11]	5 patients with bilateral TBFs 6 CIs	Severe to profound HL	1–5 years	From 1 to 2 years	PTA, speech audiometry,; promontory test, and ABR (some patients with absent responses) CT and MRI
Vermeire et al., 2012 [12]	4 patients with bilateral TBFs 4 CIs	Bilateral profound SNHL Patent cochlea and intact cochlear nerve	4 months–12 years (median value: 1–2 years)	All of them up to 12 months, and then some patients up to 5 years	PTA–speech audiometry–promontory test and vestibular tests CT and MRI
Serin et al., 2010 [13]	5 patients with bilateral TBFs 6 CIs	Bilateral profound hearing loss Intact cochlear nerve	6–36 months	Upon the 6th month, and 1st, 2nd, 3rd, 4th, 5th and 7th year	Promontory test CT and MRI
Lubner et al. [14]	12 trauma patients with TBFs 15 CIs 7 trauma patients without TBFs	Severe to profound SNHL with intact cochlear nerve	Months or years after the accident (mean value: 5.7 years)	6.5-year longitudinal follow-up with frequent sessions	PTA, promontory test (3 cases) Vestibular tests (caloric tests and VEMP) CT and MRI
Glaas et al. [15]	5 patients 6 CIs	Profound SNHL Patent cochlea and intact cochlear nerve	2–139 months (33.6+/−20.4 months)	Upon the 3rd, 6th and 12th month	PTA, promontory test, BERA, and TEOAEs Caloric vestibular testing, CT, and MRI
Khwaja et al., 2012 [16]	20 patients 16 CIs in TBF patients 6 CIs in non-TBF patients	Bilateral profound SNHL deafness Cochlear patency and intact cochlear nerve	Not reported	From 12 to 76 months (mean value: 3 yrs)	CT, MRI, and PTA Speech audiometry (BKB, CUNY, and AB tests) Promontory test Neurological/clinical examination
Alves et al., 2014 [17]	14 patients with TBFs 14 CIs 231 non- trauma patients with 231 CIs	Profound SNHL Cochlear patency	Not reported	Not reported	CT, MRI, and PTA Neuro-otological examination

**Table 3:** Audiologic/speech outcomes and complications of each study. AB: Arthur Boothroyd, ABI: auditory brainstem implant, BKB: Bamford–Kowal–Bench, BWR: bi-syllabic word recognition, C: common phrases, CID: Central Institute for the Deaf, CNC: consonant nucleus consonant, CUNY: City University of New York, FFTA: free-field tonal audiometry, FNS: facial nerve stimulation, HHIA: hearing handicap inventory for adults, NVA: Nederlandse Vereniging voor Audiologie, Plomp sentences: Dutch version of CUNY, PTA: pure-tone audiometry, SNR: signal-to-noise ratio, SR: sentence recognition, and VI: vowel identification.

Name	Follow-Up Protocol	Reported Auditory Outcomes	Complications
Lachowska et al., 2018 [7]	PTA: threshold at 0.5, 1, 2 and 4 kHz Speech audiometry: One-syllable word perception: 83% Multisyllable word perception: 98% Sentence recognition: 100% Impedance: stable values Subjective performance: all patients benefited	Auditory benefit to all patients, with significantly improved speech perception	No complications referred
Medina et al., 2014 [8]	Primary CI group VI: 90–100%—BWR: 35–100%—SR: 74–100%—C: 65–100% ABI before CI Pat. A: VI-BWR-SR not assessable Pat. B. VI: 0-BWR: 0-SR: 30%-C: 0% Pat C. VI: 0-BWR: 0-SR: 20%- CI results after auditory brainstem implants Pat A. VI:90%-BWR:90%-SR:90% Pat B VI:100%-BWR:90%-SR:69%-C 70% Pat C VI: 100%-BWR: 55%-SR: 69%-C70% Impedance telemetry: stable results Device mapping: stable findings	Auditory benefit to most patients; one patient had limited post-procedural speech performance	No complications referred
Greenberg et al., 2011 [9]	Speech perception testing (open-set sentence) Patients with older devices CID scores: 16%, 22% and 73% Newer devices scores rated from 88 to 100% and CUNY to 92% Mean score (CID): 71% Absent calorics in 16/18 ears with TBF Device mapping Rehabilitation	Auditory benefit to several recipients; a subset revealed limited speech discrimination, and some patients were not suitable candidates	2 cases of FNS (required reprogramming)
Camilleri et al., 1998 [10]	FFTA: 40–50 dB BKB sentence test in quiet: 71% VCV phoneme perception test: 67% Device mapping 6 patients benefited; 1 non-responder	Auditory benefit to most patients; variability in speech outcomes was noted	1 patient had FNS (reimplantation) 1 patient suffered wound dehiscence 2 incomplete electrode placements 1 total occlusion and placement was impossible
Hagr et al., 2011 [11]	Speech perception (open set): 2 pts (70–80%), 2 pts < 30% and the rest one 40–50% Closed set: all pts. 89–100% Subjective tests, interactivity: all patients competent users of telephone Intensive mapping: stable in all Only one patient was non-responder	Auditory benefit to most patients; one patient had limited open-set speech performance	No complications referred
Vermeire et al. 2012 [12]	PTA: 0.5–4 kHz at approximately 28 dB Speech perception evaluation in quiet: NVA monosyllabic test 34–68% Plomp sentences: 38–76%. Closed-set consonant test: 66–76% Short-vowel test: 90–100% Long vowel test: 80–100% Speech in noise: Plomp adaptive SNR (50% threshold): extremely low, 30 dB SNR Self-perceived handicap: HHIA 26–70 Mapping: stable impedance Clinical examination: no deficits	Auditory benefit to all patients, with regular device use and speech outcomes comparable to standard adult CI recipients	No complications referred
Serin et al. 2010 [13]	Speech tests at 70 dB A-weighted at 1 meter distance Closed-set sentences: initial/final phoneme test: constant improvement Open-set sentence tests: monosyllabic: >80% correct for the majority Trisyllabic: > 90% correct for the majority telephone Speech: 4/5 competent users; 1/5 after reimplantation	Auditory benefit to all patients; good speech discrimination and regular device use	1 transient facial paresis (resolved with cortisone); 1 reimplantation (fibrosis/ossification)
Lubner et al. [14]	Open-set speech perception: [CNC words]: perception was feasible Speech in noise testing: moderate improvement; worse outcomes in ossification Overall worse but insignificant outcomes for TBF cases Mapping sessions and impedance telemetry: stable values	Sustained auditory benefit at long-term follow-up; speech outcomes comparable to non-fracture recipients	1 FNS (treated with reprogramming) 1 wound dehiscence 4 partial incomplete electrode placements, 1 due to ossification
Glaas et al. [15]	Freiburg monosyllabic test: 40% at 3 months; 67% at 12 months Freiburg numbers test: acceptable proportion Impedance verification: stable impedances	Auditory benefit to all recipients; speech discrimination improved over time	1 FNS (reprogrammed successfully)
Khwaja S. et al., 2012 [16]	BKB: similar for both groups (to noise and silence) 68% at 9 months and 64% at the last control CUNY: similar for both groups 86% at 9 months and 83% at the last control AB: similar for both groups 59% at 9 months and 60% at last control. Impedance and stimulation control	Highly variable outcomes; auditory benefit to many patients, with a subset demonstrating poor speech discrimination or limited device use	3 cases of FNS 1 reimplantation 1 partial insertion due to cochlear ossification Cognitive deficits hindered proper mapping
Alves M. et al., 2014 [17]	PTA: trauma pts: 33.3 ±6.85 dB at 500 Hz, 33 ± 5.36 dB at 1000 Hz, and 35.8 ± 2.88 dB Non-trauma pts: 500 Hz at 31.6 ± 8.11 dB, 1000 Hz at 31.4 ±7.74 dB, and 4000 Hz at 33.2 ± 6.62 dB Speech audiometry: similar to control group Consonant discrimination test: poorer outcome for TBF group (40% vs. 60%) 100-word open-set sentences test (face-to- face): no intergroup difference 100-word open-set sentences test (telephone): poorer for TBF group (35.8% vs. 60.7%) Monosyllable recognition test: poorer outcome in TBF group (52% vs. 67%) Rehabilitation	Significant auditory benefit to most post-traumatic recipients, although average speech scores were lower compared with non-trauma causes	None referred

## Data Availability

No new data were created.

## Supplementary Materials

The following supporting information can be downloaded at https://www.mdpi.com/article/10.3390/brainsci16020227/s1. Supplementary Material S1. PRISMA 2020 Check List. Supplementary Materials S2. Complete electronic search procedures

## Abbreviations

The following abbreviations were used in this manuscript:

AB	Arthur Boothroyd
ABI	Auditory Brainstem
ABR	Auditory Brainstem Responses
BAHA	Bone-Anchored Hearing Aids
BERA	Brainstem-Evoked Response Audiometry
BKB	Bamford–Kowal–Bench
BWR	Bi-Syllabic Word Recognition
C	Common Phrases
CIs	Cochlear Implants
CID	Central Institute for the Deaf
CNC	Consonant Nucleus Consonant
CT	Computed Tomography
CUNY	City University of New York
ENG	Electronystagmography
FFTA	Free-Field Tonal Audiometry
FN	Facial Nerve
FNS	Facial Nerve Stimulation
HHIA	Hearing Handicap Inventory for Adults
HL	Hearing Loss
HRCT	High-Resolution Computed Tomography
JBI	Joanna Briggs Institute
MRI	Magnetic Resonance Imaging
NVA	Nederlandse Vereniging voor Audiologie
PTA	Pure-Tone Audiometry
SNHL	Sensorineural Hearing Loss
SNR	Signal-to-Noise Ratio
SR	Sentence Recognition
TEOAEs	Transmission-Evoked Otoacoustic Emissions
VI	Vowel Identification
