# Supplementary material for: Cochlear Implantation After Temporal Bone Fracture: A Systematic Review of Preoperative Predictors and Timing
Source: Brain Sci. 2026 Feb 14;16(2):227. doi: 10.3390/brainsci16020227 (PMC12938646; doi:10.3390/brainsci16020227)
Supplement: Supplementary file 1 [file brainsci-16-00227-s001.zip › brainsci-4118547-Supplementary Materials/brainsci-4118547-Supplementary Materials S2-Complete electronic search procedures.docx]

**Supplementary Materials S2**

**S2.1 Pubmed/Medline search string**

**Platform:** NCBI PubMed

**Date of last search:** 30 September 2025

("Hearing Loss"[Mesh] OR "Hearing Loss, Sensorineural"[Mesh] OR "Deafness"[Mesh]

OR "hearing loss"[Title/Abstract] OR "sensorineural hearing loss"[Title/Abstract])

AND

("Head Injuries, Closed"[Mesh] OR "Craniocerebral Trauma"[Mesh]

OR "Traumatic Brain Injury"[Mesh]

OR "Temporal Bone"[Mesh]

OR "Temporal Bone Fractures"[Title/Abstract]

OR "temporal bone fracture"[Title/Abstract]

OR "head trauma"[Title/Abstract])

AND

("Cochlear Implants"[Mesh] OR "Cochlear Implantation"[Title/Abstract]

OR "cochlear implant"[Title/Abstract]

OR "hearing aids"[Mesh])

Filters applied: Humans; English language

Coverage: December 1995–September 2025

**S2.2 Scopus Search String**

**Platform:** Scopus (Elsevier)
**Date of last search:** 30 September 2025

(TITLE-ABS-KEY("hearing loss" OR "sensorineural hearing loss" OR deafness))

AND

(TITLE-ABS-KEY("temporal bone fracture" OR "head trauma" OR "traumatic brain injury"))

AND

(TITLE-ABS-KEY("cochlear implant" OR "cochlear implantation" OR "hearing aid"))

AND

(LIMIT-TO(LANGUAGE, "English"))

AND

(LIMIT-TO(DOCTYPE, "ar"))

**S2.3 Google Scholar Search String**

**Platform:** Google Scholar
**Date of last search:** 30 September 2025

"temporal bone fracture" "cochlear implant" "hearing loss"

**Screening methodology:**

- Results sorted by relevance
- First 200 results screened (titles/abstracts)
- English-language human studies included
- December 1995–September 2025
